# Diverse immunotherapies can effectively treat syngeneic brainstem tumors in the absence of overt toxicity

**DOI:** 10.1186/s40425-019-0673-2

**Published:** 2019-07-17

**Authors:** Matthew R. Schuelke, Phonphimon Wongthida, Jill Thompson, Timothy Kottke, Christopher B. Driscoll, Amanda L. Huff, Kevin G. Shim, Matt Coffey, Jose Pulido, Laura Evgin, Richard G. Vile

**Affiliations:** 1 Department of Immunology, Mayo Clinic, Rochester, MN 55905 USA; 2 Medical Scientist Training Program, Mayo Clinic, Rochester, MN 55905 USA; 3 Department of Molecular Medicine, Mayo Clinic, Rochester, MN 55905 USA; 4 Virology and Gene Therapy Track, Mayo Clinic, Rochester, MN 55905 USA; 5 Oncolytics Biotech, Inc., Calgary, AB T2N 1X7 Canada; 6 Department of Ophthalmology, Mayo Clinic, Rochester, MN 55905 USA; 7 Leeds Cancer Research UK Clinical Centre, Faculty of Medicine and Health, St James’ University Hospital, University of Leeds, West Yorkshire, UK

**Keywords:** Immunotherapy, Brainstem, Toxicity, DIPG

## Abstract

**Background:**

Immunotherapy has shown remarkable clinical promise in the treatment of various types of cancers. However, clinical benefits derive from a highly inflammatory mechanism of action. This presents unique challenges for use in pediatric brainstem tumors including diffuse intrinsic pontine glioma (DIPG), since treatment-related inflammation could cause catastrophic toxicity. Therefore, the goal of this study was to investigate whether inflammatory, immune-based therapies are likely to be too dangerous to pursue for the treatment of pediatric brainstem tumors.

**Methods:**

To complement previous immunotherapy studies using patient-derived xenografts in immunodeficient mice, we developed fully immunocompetent models of immunotherapy using transplantable, syngeneic tumors. These four models – HSVtk/GCV suicide gene immunotherapy, oncolytic viroimmunotherapy, adoptive T cell transfer, and CAR T cell therapy – have been optimized to treat tumors outside of the CNS and induce a broad spectrum of inflammatory profiles, maximizing the chances of observing brainstem toxicity.

**Results:**

All four models achieved anti-tumor efficacy in the absence of toxicity, with the exception of recombinant vaccinia virus expressing GMCSF, which demonstrated inflammatory toxicity. Histology, imaging, and flow cytometry confirmed the presence of brainstem inflammation in all models. Where used, the addition of immune checkpoint blockade did not introduce toxicity.

**Conclusions:**

It remains imperative to regard the brainstem with caution for immunotherapeutic intervention. Nonetheless, we show that further careful development of immunotherapies for pediatric brainstem tumors is warranted to harness the potential potency of anti-tumor immune responses, despite their possible toxicity within this anatomically sensitive location.

## Background

Brain tumors are the leading cause of pediatric cancer death [1]. Ten to 15 % of these tumors occur in the brainstem, most of which are a uniformly fatal disease classified as an H3K27M-mutant diffuse midline glioma, or historically, diffuse intrinsic pontine glioma (DIPG) [2, 3]. Radiation extends survival by several months and dexamethasone is used for symptomatic control, but unfortunately, decades of clinical research have not increased median overall survival beyond 9–11 months [2].

Cancer immunotherapy is among the most promising areas of biomedical research, with recent FDA approval of the first chimeric antigen receptor (CAR) T cell [4] and oncolytic virus [5] therapies. In addition to strong anti-tumor activity, these treatments have the potential to provide long-term cancer immunosurveillance through the generation of immunologic memory [6, 7]. Furthermore, while traditional chemotherapy and radiation have long-term, developmental effects on pediatric patients [8], clinical studies in adults suggest that immunotherapies may have favorable long-term safety profiles [9, 10], though ongoing studies are validating this in children.

However, brainstem gliomas like DIPG provide unique challenges for immunotherapy. Physiologically, the brainstem controls vital functions [11], requiring that therapies avoid damage to healthy tissue. Infectious or autoimmune inflammation of the brainstem carries high morbidity and mortality [12], raising concerns for cancer immunotherapies in this location. Additionally, DIPGs contain few adaptive immune cells, suggesting a lack of functional immunosurveillance and a need for induction of de novo immune responses [13, 14].

These data raise the question of whether potentially curative immunotherapy for brainstem tumors may generate unacceptable toxicity. Preclinically, cancer vaccines [15] and CAR T cells [16] have been studied in DIPG xenograft models, establishing evidence of anti-tumor efficacy. However, while these human xenograft models allow for proof-of-concept efficacy, exploration of immune-mediated toxicity and associated inflammation is best recapitulated in an immunocompetent animal model. To this end, we used previously-validated syngeneic gliomas and melanomas to establish brainstem tumor models to assess a diverse range of immunotherapies. Our goal was not to compare relative efficacies of each therapy, but rather to ascertain the potential for immune-mediated toxicity in the brainstem. From these studies, we demonstrate that although the possibility of therapy-related inflammatory toxicity exists, a diverse range of immunotherapies can extend survival of mice bearing syngeneic brainstem tumors without generating overt neurologic toxicity. These results suggest greater consideration of clinical immunotherapy trials for pediatric brainstem tumors.

## Materials and methods

### Cell lines and viruses

GL261 and GL261-QUAD cells were obtained from Dr. Aaron Johnson (Mayo Clinic). GL261-QUAD cells were originally created by John Ohlfest, et al [17], and stably express the model tumor antigens chicken OVA_257–264_, chicken OVA_323–339_, human gp100_25–33_, and the mouse alloantigen I-Ea_52–68_. Both GL261 tumor lines were grown in DMEM (HyClone, Logan, UT, USA) + 10% FBS (Life Technologies, Carlsbad, CA). B16.F1 parental murine melanoma cells were obtained from the ATCC (Manassas, VA). B16tk cells were derived from a B16.F1 clone stably infected with a lentivirus expressing the Herpes Simplex Virus thymidine kinase (HSV-1 TK) gene. Following stable selection in 1.25 μg/mL puromycin, these cells were shown to be sensitive to Ganciclovir (APP Pharmaceuticals, Barceloneta, PR) at 5 μg/ml. B16tk cells were grown in DMEM (HyClone, Logan, UT, USA) + 10% FBS (Life Technologies, Carlsbad, CA) + 1.25 μg/mL puromycin (Sigma, St. Louis, MO) until challenge. B16-ova cells were B16.F1 cells transfected with pcDNA3.1OVA and maintained in 10% DMEM with 5 mg/mL G418 selection media. The B16-EGFRvIII cell line was generated by retroviral transduction with pBABE PURO encoding the murine EGFRvIII modified by the deletion of 500 aa from the intracellular domain of the protein using a construct given as a kind gift from Dr. Luis Sanchez-Perez and Dr. John Sampson (Duke University, Durham, NC) [18]. A clonally derived cell line was subsequently maintained in 1.25 μg/mL of puromycin.

Cell lines were authenticated by morphology, growth characteristics, PCR for melanoma specific gene expression (gp100, TYRP-1 and TYRP-2) and biologic behavior, tested mycoplasma-free, and frozen. EGFRvIII-positive cells were verified by flow cytometry staining with an anti-EGFRvIII primary antibody (L8A4). Cells were cultured less than 3 months after thawing. Cells were tested for mycoplasma using the MycoAlert Mycoplasma Detection Kit (Lonza Rockland, Inc. ME, USA).

For recombinant vaccinia virus (VV) production, CV-1 cells were infected by vaccinia virus (WR strain) and transfected with the pSC65 plasmid transfer vector (a generous gift from Dr. Bernard Moss, NIAID) containing murine granulocyte monocyte colony stimulating factor (GM-CSF) cDNA [19]. Recombinant viruses were isolated and then bulked up in Hela cells (ATCC, Manassass, VA), followed by sucrose cushion purification. Purified virus was titered on Hela cells and stored at -80C. Clinical-grade reovirus was acquired from Oncolytics Biotech (Calgary, Canada).

GL261 cells were infected with reovirus or VV-GMCSF at an MOI of 10 followed by exposure to Cell Titer Blue (Promega) for survival assessment or harvesting for replication assessment using a plaque assay on Hela cells (vaccinia) or L929 cells (reovirus).

All vesicular stomatitis viruses (VSV) were generated as previously described [20]. Briefly, VSV (Indiana serotype) expressing human gp100 or chicken ovalbumin was generated by cloning the respective antigen into the pVSV-XN2 plasmid by inserting between the VSV G and L proteins. VSV was titered by plaque assay on BHK cells and stored at -80C.

### Mice

Female C57BL/6 mice at 6–8 weeks of age (Jackson Labs) were used for all in vivo experiments. OT-I [21] and pmel [22] mice were bred at the Mayo Clinic, and harvested between 8 and 14 weeks of age for adoptive transfer experiments.

### Transgenic T cell preparation

Pmel or OT-I T cells were harvested from transgenic pmel or OT-I mouse spleens, respectively, and underwent a magnetic bead negative sort for CD8+ cell isolation (Miltenyi Biotec).

### CAR T cell preparation

EGFRvIII-reactive chimeric antigen receptor construct was obtained as a kind gift of Dr. Steven Feldman (National Cancer Institute, Bethesda, MD), and CAR T cells were produced as previously described [23]. In brief, mouse splenocytes were harvested from C57BL/6 mice and activated in IL2 (50 U/mL) and ConA (2.5 μg/mL) for two days. Cells were transduced with a retroviral construct expressing an EGFRvIII-reactive scFv followed by CD3zeta, CD28, and 4-1BB murine intracellular signaling domains, followed by an IRES and either a luciferase or GFP tag. CAR T cells were harvested three days later for experimental use.

### In vivo studies

C57BL/6 mice were challenged with brainstem tumors via stereotactic implantation using established coordinates [24]. Mice were monitored daily for gross neurologic symptoms including gait abnormalities, hunching, lethargy, seizures, paralysis, circling, and head tilt. Upon presentation of gross neurologic symptoms or poor body conditioning, mice were euthanized in accordance with IACUC standards.

For suicide gene therapy studies, mice bearing B16tk brainstem tumors were treated on days 4–8 and 11–15 with ganciclovir (GCV) (50 mg/kg i.p.) (APP Pharmaceuticals). Dexamethasone co-treatment (1.0 mg/kg i.p.) (Fresenius Kabi) began on day 4 post-tumor implantation and continued for the remainder of the experiment. For radiation studies, mice received 10Gy of whole brain irradiation using a Cesium-137 irradiator on day 4 post tumor implantation, followed by GCV on days 6–10 and 13–17.

For oncolytic virotherapy, GL261 glioma cells were suspended in VV-GMCSF or reovirus at an MOI of 10 (5 × 10^5^ pfu) immediately prior to implantation. Ten-day established tumors were treated with reovirus or PBS stereotactic injection. Mice were also treated with anti-CTLA4 (100 μg/mouse i.p.) (9D9, BioXCell) and anti-PD1 (250 μg/mouse i.p.) (RMP1–14, BioXCell) antibodies or an IgG control (350 μg/mouse i.p.) (Jackson ImmunoResearch) on days 10, 13, and 16.

For adoptive cell transfer, mice bearing B16-ova brainstem tumors were treated with pmel T cells on day 6 (1 × 10^6^ cells i.v.), and VSV-hgp100 4–6 h later (5 × 10^6^ pfu i.v.). VSV-hgp100 was readministered on days 6 and 8. This protocol was repeated using GL261-QUAD gliomas, OT-I T cells, and VSV-ova.

For CAR T cell studies, three days after B16-EGFRvIII tumor challenge, mice received 5Gy of total body irradiation using a Cesium-137 irradiator. One day later, mice received EGFRvIII-CAR T cells or untransduced controls (1 × 10^7^ cells IV). Three hours later, mice were administered anti-PD1 antibody (250 μg/mouse i.p.) (RMP1–14, BioXCell) or an IgG control (250 μg/mouse i.p.) (Jackson ImmunoResearch). Anti-PD1 treatment was repeated on days 7 and 10.

### In vivo MRI and bioluminescence imaging

T1 and T2 MRI images were acquired using a Bruker DRX-300 (300 MHz 1H) 7-Tesla vertical bore small animal imaging system (Bruker Biospin) as described [25]. Analyze 11.0 software (Biomedical Imaging Resource, Mayo Clinic) was used by blinded reviewers for image analysis. Bioluminescence imaging was performed on CAR T cell-treated mice using an IVIS Spectrum system (Xenogen Corp.) [25].

### Flow cytometry

Brains for flow cytometry analysis were prepared using dounce homogenization and a Percoll gradient solution as previously described [26]. Enriched immune cells were stained using the following antibodies: CD45 (30-F11 or A20), Thy1.1 (HIS51), CD4 (GK1.5), CD8 (53–6.7), GR1 (RB6-8C5), CD11b (M1/70), CD11c (HL3), NK1.1 (PK136), CD19 (6D5), I-A/I-E (MHCII) (M5/114.15.2), and fixable live dead viability dye (Zombie NIR).

VITAL killing assay was performed as previously described [27]. Briefly, B16-EGFRvIII targets or parental B16 non-target cells were stained with CellTrace Violet (Molecular Probes, Eugene, OR) or CellTrace CFSE (Molecular Probes, Eugene, OR) prior to plating at a 1:1 ratio. CAR T cells were then co-incubated for 24 h, followed by fixable live dead staining with Zombie NIR (Biolegend, San Diego, CA). The target:nontarget ratio at various effector:target ratios was used to calculate specific killing.

For ex vivo T cell restimulation, spleens from pmel/VSVhgp100-treated mice were made into a single cell suspension and plated. hgp100_25–33_ (cognate pmel antigen) or SIINFEKL (ovalbumin antigen) were added at 5 uM to the prepared splenocytes and incubated at 37C. Two hours later, brefeldin A (Cytofix/Cytoperm Plus, BD, Franklin Lakes, NJ) was added, followed by 4 more hours of incubation. At that time, flow cytometry surface staining was performed using the following antibodies: anti-Thy1.1 (HIS51), anti-CD4 (GK1.5), anti-CD8 (53–6.7), and fixable live dead viability dye (Zombie NIR). Cells were then permeabilized and intracellularly stained with: anti-IFNg (XMG1.2), anti-TNFa (MP6-XT22), and anti-IL2 (JE56-5H4).

### Histology and immunofluorescence

All tissues were fixed in 10% formalin, sectioned, and embedded in paraffin or underwent H&E staining (Mayo Clinic Histology Core Facility). Immunofluorescence staining was performed based upon established protocols [28]. Briefly, slides were deparaffinized in a series of washes of decreasing ethanol content. CD3, GFP, CD11b-stained slides underwent heat-mediated antigen retrieval using sodium citrate buffer, while VV-stained slides used Tris/EDTA buffer. Slides were then stained with anti-CD3 (ab16669, Abcam, Cambridge, MA), anti-VV (ab35219, Abcam, Cambridge, MA), anti-GFP (ab6556, Abcam, Cambridge, MA), or anti-CD11b (ab133357, Abcam, Cambridge, MA) antibodies, followed by secondary staining with an AF568-tagged goat anti-rabbit antibody (A11011, Invitrogen, Carlsbad, CA) and counterstaining with DAPI. Images were acquired with an LSM780 confocal microscope and Zen software (Carl Zeiss, Thornwood, NY). Quantification was performed using ImageJ for tumor area calculation and blinded manual counting of CD3+ cells.

### Statistics

Mantel-Cox Log-Rank test with Holm-Bonferroni correction for multiple comparisons was used to analyze Kaplan-Meier survival curves. Student’s T tests with Holm-Bonferroni correction for multiple comparisons were used for in vitro and ex vivo analysis where appropriate. Statistical significance was set at *p* < 0.05 for all experiments. All analysis was performed within GraphPad Prism 6 software.

## Results

### Suicide gene therapy

**Fig. 1:**
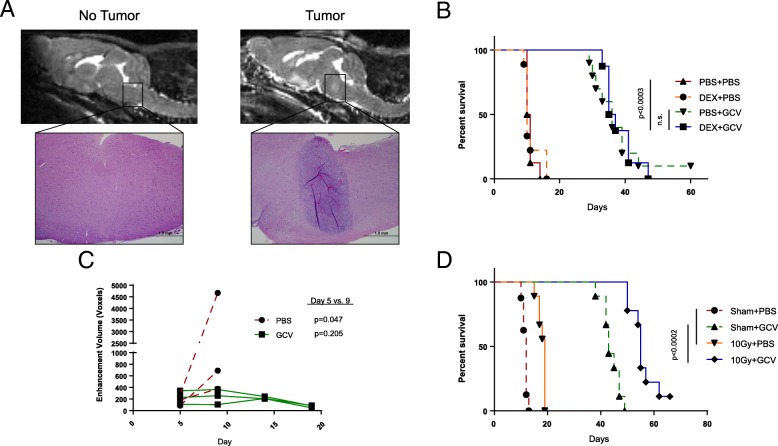
Suicide gene therapy effectively treats brainstem tumors without overt toxicity. (**a**) B16tk cells (right) or PBS (left) were stereotactically implanted into the brainstem of C57BL/6 mice that underwent T2 MRI imaging on Day 9. Inset H&E images at 4x. (**b**) Kaplan-Meier survival curve for B16tk tumors treated with two five-day courses of GCV and daily dexamethasone. (*n* = 10 mice/group) Figure is representative of two independent experiments. (**c**) Tumor volume assessment by T1 MRI imaging of treated and untreated mice. (*n* = 3 mice/group) Paired t-test of the difference in tumor volumes between Days 5 and 9 for each group. (**d**) Kaplan-Meier survival curve for B16tk tumors treated with 10Gy whole brain irradiation and GCV. (*n* = 9 mice/group) Figure is representative of one experiment

We and others have shown that suicide gene therapy acts through an inflammatory mechanism dependent upon immune effectors [29, 30]. To model this therapeutic strategy, we used published brainstem coordinates [24] to stereotactically implant B16 murine melanomas stably expressing herpes simplex virus thymidine kinase (B16tk), which are sensitive to ganciclovir (GCV) prodrug treatment. Although melanomas do not frequently metastasize to the brainstem, this model was well-characterized in our laboratory and maintained high expression of the suicide gene, maximizing the potential for therapeutic toxicity. Similar to aggressive brainstem gliomas, untreated mice succumbed to disease within 10–15 days, with tumor location confirmed by MRI and histology (Fig. 1A). When treated with GCV, mice survived significantly longer than untreated controls (Fig. 1B**)** with decreased tumor growth (Fig. 1C). Importantly, gross neurologic examination during GCV treatment did not reveal any deficits or other signs of therapy-related toxicity. To assess compatibility with clinical treatments, we combined suicide gene therapy with dexamethasone or radiation, both known immunomodulators [31, 32]. Daily concurrent administration of dexamethasone did not decrease GCV treatment efficacy (Fig. 1B). Additionally, pretreatment with 10Gy of whole brain radiation increased survival beyond PBS-treated mice, and radiation enhanced GCV therapy (Fig. 1D).

**Fig. 2:**
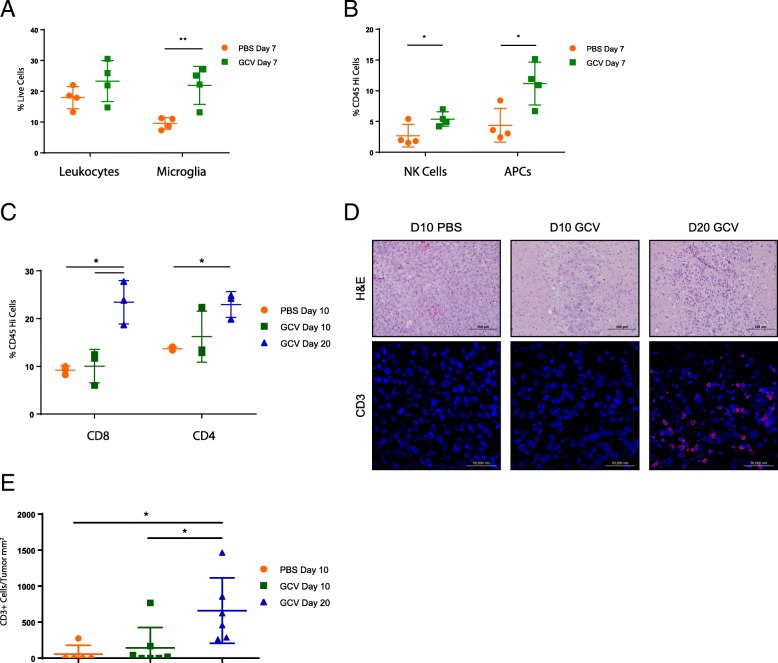
Suicide gene therapy generates brainstem inflammation. (**a,b,c**) Whole brain flow cytometry of mice bearing B16tk brainstem tumors treated with GCV or PBS from Days 4–8 and 10–14 post-tumor implantation. Leukocytes were gated as CD45^Hi^; Microglia were gated as CD45^Mid^, CD11b+; APCs were gated as CD45^Hi^ and either CD11b+, CD11c + or MHCII+. * *p* ≤ 0.05 (*n* = 3–4 mice/group) (**d**) Representative histology from (**c**). H&E (20x, top) and anti-CD3 (red)/DAPI (blue) (40x, bottom), with quantitation in (**e**). * *p* < 0.05

Whole brain flow cytometry of GCV-treated mice three days after treatment initiation demonstrated an increase in NK cells, antigen presenting cells, and microglia (Fig. 2A,B), suggesting innate immune activation in response to suicide gene-induced cell death. Thirteen days later, this led to an increase in CD4 and CD8+ T cell infiltration in GCV-treated mice (Fig. 2C). No change was observed six or thirteen days after treatment in CD45^Hi^ cells, or B cells, NK cells, dendritic cells, or macrophages within that population (not shown). Immunofluorescence staining confirmed an increase in CD3+ mononuclear infiltrates (Fig. 2D,E). These data show that directly-cytotoxic, inflammatory therapies such as suicide gene therapy can effectively treat brainstem tumors in the absence of overt toxicity.

### Oncolytic viroimmunotherapy

**Fig. 3:**
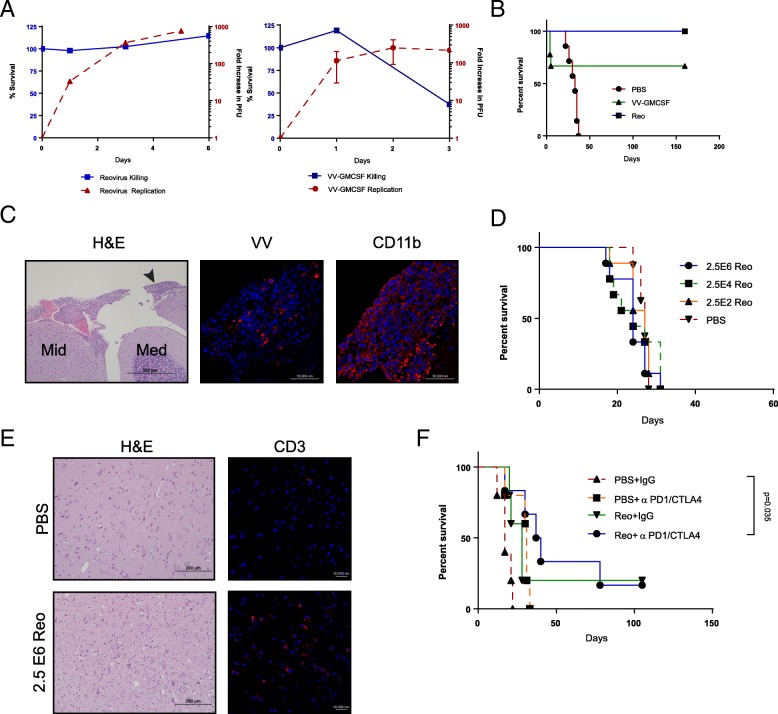
Immunostimulatory oncolytic virotherapy in the brainstem demonstrates both inflammatory toxicity and anti-tumor efficacy. (**a**) GL261 cells were infected in vitro with VV-GMCSF or reovirus (MOI 10). Cell survival (left y-axis, solid bar) or viral titers (right y-axis, dashed bar). (**b**) Kaplan-Meier survival curve for mice receiving VV-GMCSF/GL261, Reo/GL261, or PBS/GL261 co-implantation. (n = 9 mice/group) Figure is representative of two independent experiments. (**c**) Representative mouse euthanized for neurologic symptoms four days after VV-GMCSF and tumor co-implantation. H&E image (left, 10x); midbrain (Mid) and medulla (Med), with black arrowhead to indicate inset meningeal infiltrate (vaccinia antigen, red, 40x, middle; CD11b, red, 40x, right). (**d**) Kaplan-Meier survival curve for 10-day established GL261 tumors treated intratumorally with escalating doses of reovirus. (*n* = 9 mice/group) Figure is representative of three independent experiments. (**e**) Representative mice euthanized seven days after reovirus or PBS treatment for histologic analysis. H&E image (left, 20x) and anti-CD3 (red)/DAPI (blue) (right, 40x). (**f**) Kaplan-Meier survival curve for 10-day established GL261 tumors treated with reovirus (2.5E6 pfu) or PBS, followed by anti-CTLA4/anti-PD1 therapy. (*n* = 5–6 mice/group) Figure is representative of two independent experiments

To test highly inflammatory therapies, we selected two viruses with differing replication kinetics and immunogenicities. The first was serotype-3 Dearing strain reovirus with immune-dependent anti-tumor efficacy previously used in clinical trials to treat glioblastoma [33, 34]. The second virus was an attenuated, highly-inflammatory vaccinia virus expressing granulocyte-monocyte colony stimulating factor (VV-GMCSF), which has been studied previously in gliomas and generates an innate and adaptive anti-tumor immune response [35, 36]. Both viruses replicated in GL261 cells in vitro, although only vaccinia virus killed cells within one week (Fig. 3A). To model maximal viral replication, killing, and inflammatory toxicity, GL261 cells were mixed with each virus immediately prior to implantation into the brainstem of C57 mice. Within several days, mice receiving VV-GMCSF began losing weight and exhibited overt neurologic symptoms such as hunching, lethargy, and ataxia, with 33% of mice requiring euthanasia (Fig. 3B). A CD11b + meningeal cellular infiltrate was observed in regions also positive for vaccinia antigen (Fig. 3C). Interestingly, mice surviving initial VV-GMCSF-related toxicity were tumor-free after 150 days (Fig. 3B). Reovirus-treated mice did not develop neurologic symptoms requiring euthanasia, but similarly remained tumor-free for 150 days (Fig. 3B). Because of its ability to prevent tumor development in the absence of toxicity, we tested reovirus against established GL261 brainstem tumors. Surprisingly, injection of GL261 brainstem tumors on Day 10 with escalating doses of reovirus neither improved survival over PBS-injected controls, nor demonstrated any toxicity (Fig. 3D). In a corollary experiment, mice were sacrificed seven days after viral administration for histological examination of CD11b and CD3 expression. While CD11b + cells were not observed (not shown), CD3+ cells were present in the brainstem of Reovirus-treated mice (Fig. 3E), suggesting that while T cells were recruited to the brainstem, their activity may have been inhibited. Based on previous studies in gliomas [37, 38], we hypothesized that immunosuppressive factors may be restricting reovirus’ ability to elicit an anti-tumor immune response. To overcome this, we tested anti-PD1 and anti-CTLA4 immune checkpoint blockade to increase the potential for additive inflammatory toxicity or enhanced efficacy. We did not observe any overt toxicity in mice treated with either ICB alone or in combination with reovirus. However, while neither ICB nor reovirus significantly extended survival as monotherapies, combination therapy led to enhanced survival over sham-treated controls (*p* = 0.035 after correction for multiple comparisons) (Fig. 3F). These data suggest that although there is potential for inflammatory toxicity, direct administration of certain viruses can treat brainstem tumors, especially in combination with immune checkpoint blockade.

### Adoptive T cell transfer therapy

**Fig. 4:**
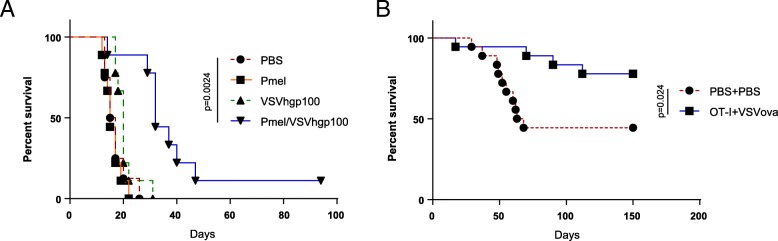
Transgenic T cell therapy extends survival in mice bearing syngeneic brainstem tumors. (**a**) Kaplan-Meier survival curve for B16-ova tumors were treated with PBS or pmel T cells, followed 4–6 h later by PBS or VSV-hgp100 and two follow-up doses of VSV-hgp100 or PBS. (*n* = 9 mice/group) Figure is representative of three independent experiments. (**b**) Kaplan-Meier survival curve for mice bearing GL261-QUAD tumors treated with PBS or OT-I T cells, followed 4–6 h later by PBS or VSV-ova and two follow-up doses of VSV-ova or PBS. (*n* = 18 mice/group) Figure is representative of one experiment

Next, we tested whether an anti-tumor, T cell-mediated therapy could be tolerated in the brainstem. We have previously shown that naïve transgenic T cells transferred into mice can be activated in vivo by viral expression of their cognate antigens, leading to regression of flank tumors or metastases [39–41]. Mice bearing brainstem B16 melanomas were treated with naïve pmel transgenic T cells recognizing the murine melanoma antigen gp100, followed by three doses of VSV expressing human gp100 (VSV-hgp100), a known heteroclitic activator of pmel T cells [39, 41]. Additionally, these tumors expressed the model antigen ovalbumin (ova), for subsequent evaluation of antigen spread in the endogenous T cell compartment. When treated with either pmel T cells or VSV-hgp100 alone, mice succumbed to disease alongside untreated controls (Fig. 4A). However, treatment with both therapies significantly extended median survival by 16 days compared to untreated controls. To establish that this effect was neither antigen nor tumor specific, we repeated these studies using a GL261-QUAD murine glioma model [17], OT-I T cells, and VSV expressing ovalbumin (VSV-ova). Although this tumor’s immunogenicity caused it to be spontaneously rejected in half of the mice, OT-I T cell and VSV-ova treatment did not elicit any inflammatory toxicity, and treated mice survived significantly longer than untreated controls (Fig. 4B).

**Fig. 5:**
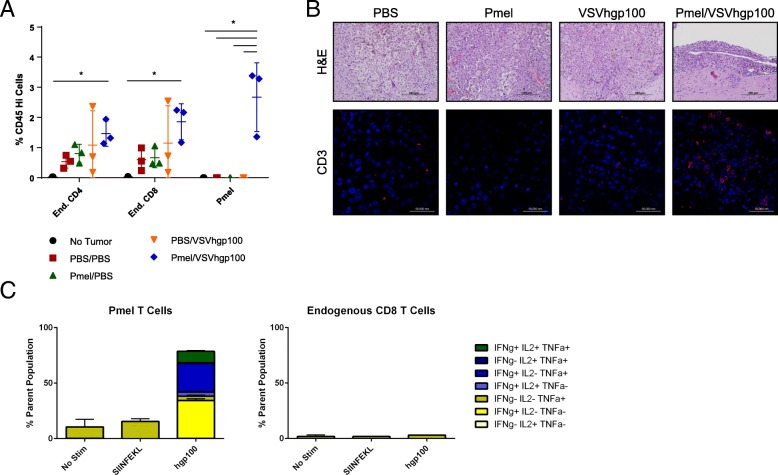
Transgenic T cells traffic to the brainstem and recruit endogenous immune cells. (**a**) Whole brain flow cytometry of mice treated as described in Fig. 4A. Analysis performed on Day 15 post-tumor implantation. * *p* ≤ 0.05 (*n* = 3 mice/group) (**b**) Representative histology from (**a**). H&E (20x, top) and anti-CD3 (red)/ DAPI (blue) (40x, bottom). (**c**) Splenocytes from mice treated with pmels and VSV-hgp100 and harvested in (**a**) and (**b**) were incubated for 6 h with vehicle, hgp100_25–33_, or SIINFEKL (ovalbumin immunogenic peptide), and underwent intracellular staining for cytokines. (*n* = 3 mice/group)

Whole brain flow cytometry of mice treated with pmel T cells and VSV-hgp100 showed a significant increase in pmel (Thy1.1+) T cells three days after the last VSV dose, and a trend towards significance for endogenous (Thy1.1-) CD4 and CD8 T cells compared to monotherapy or untreated mice (Fig. 5A). To confirm T cell brainstem infiltration, immunofluorescence staining demonstrated an increase in CD3+ mononuclear cells localized to the tumor in pmel T cell/VSV-hgp100-treated mice (Fig. 5B). Importantly, splenic pmel T cells from treated mice secreted inflammatory cytokines ex vivo in response to their cognate antigen, though endogenous CD8 T cells were unresponsive to either the vaccinated antigen (hgp100) or a tumor antigen (ovalbumin-derived SIINFEKL) (Fig. 5C). Together, these data suggest that T cell therapy with activated anti-tumor T cells generates brainstem infiltrates that exert anti-tumor activity without overt evidence of toxicity.

### Chimeric antigen receptor (CAR) T cell therapy

**Fig. 6:**
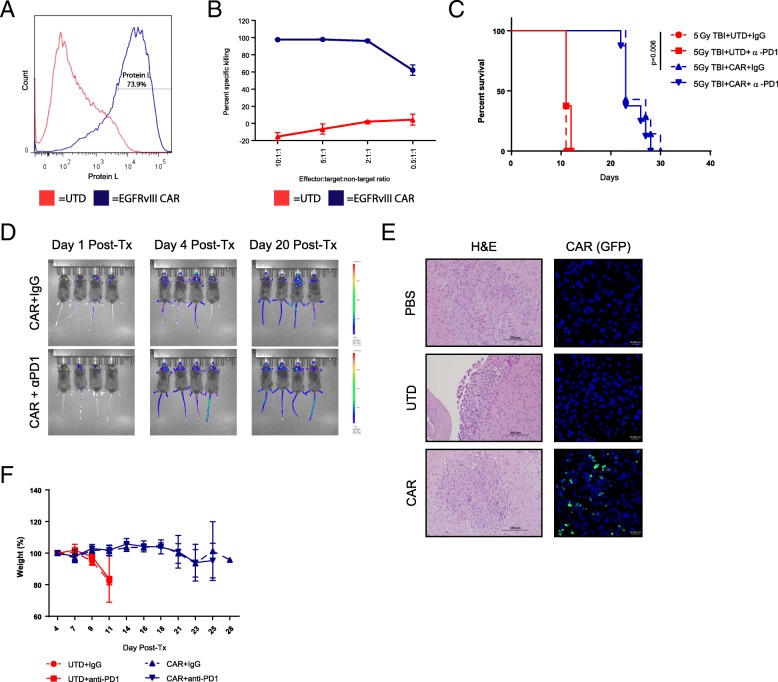
CAR T cells traffic to the brainstem and extend survival without overt toxicity. (**a**) EGFRvIII-CAR transduction quantification using biotinylated protein L and streptavidin-PE staining. (**b**) VITAL assay of B16-EGFRvIII-specific killing over B16-parental cells by EGFRvIII-CAR T cells after 24 h. (*n* = 2) (**c**) Kaplan-Meier survival curve of B16-EGFRvIII brainstem tumors treated with 5Gy total body irradiation followed by EGFRvIII-CAR T cells or untransduced controls and anti-PD1 antibody or IgG control. (*n* = 8 mice/group) Figure is representative of two independent experiments. (**d**) Representative IVIS bioluminescent images of EGFRvIII-CAR-Luciferase treated mice. (**e**) Representative mice bearing B16-EGFRvIII brainstem tumors sacrificed for histologic analysis four days after GFP-tagged CAR administration. H&E (left, 20x) and anti-GFP (green)/DAPI(blue) (right, 40x) (**f**) Serial weight measurements from (**c**). (*n* = 8 mice/group)

To model a clinically-relevant T cell immunotherapy, we used a retroviral, third-generation murine CAR construct targeting epidermal growth factor receptor variant III (EGFRvIII), a commonly mutated receptor in glioblastoma [42]. Although EGFRvIII is not expressed by pediatric brainstem tumors, it served as a model antigen in this context. This construct successfully transduced murine splenocytes to more than 70% CAR positivity (Fig. 6A), with the resulting CAR T cells successfully and selectively killing B16 murine melanomas stably expressing EGFRvIII (B16-EGFRvIII) in the presence of parental B16 cells in vitro (Fig. 6B). Subsequently, B16-EGFRvIII cells were implanted into the brainstem, and following 5Gy total body irradiation (TBI) for partial lymphodepletion [42], EGFRvIII-CAR, luciferase-tagged T cells were administered intravenously. Mice receiving TBI and EGFRvIII-CAR T cells survived significantly longer than mice receiving untransduced T cells (UTD) (Fig. 6C). However, anti-PD1 checkpoint blockade did not significantly impact survival, but also did not induce toxicity. Using IVIS imaging, CAR T cells were visualized in the brainstem as early as one day post-administration (Fig. 6D), which was histologically confirmed using GFP-tagged CAR T cells (Fig. 6E). During this therapeutic window where T cells were present in the brainstem, mice receiving CAR T cells did not exhibit any neurologic symptoms or weight loss (Fig. 6F). Our data here suggest that CAR T cell therapy, which is currently approved for CD19+ liquid malignancies, may have a favorable safety profile in brainstem tumors. However, these murine models provide limited opportunity to evaluate toxicities experienced in human trials, due to the highly specific nature of each CAR target.

## Discussion

The goal of this study was to investigate whether inflammatory, immune-based therapies are likely to be too dangerous for the treatment of pediatric brainstem tumors. While cancer immunotherapy has shown remarkable clinical promise, these benefits derive from a highly inflammatory mechanism of action involving both innate and adaptive immune effector cells. Tumors growing in the brainstem present largely unique clinical challenges in this respect because of the potential for life-threatening inflammation-driven swelling. Hence, whereas pseudoprogression is now recognized as a clinically beneficial sign of immunotherapeutic success [43], the recruitment of such immune effectors into the space-limited context of a tumor in the brainstem may be more dangerous than the tumor itself. Therefore, our goal was to test the hypothesis that inflammatory therapies would be catastrophically toxic for the treatment of brainstem tumors.

To this end, we used stereotactic implantation to establish syngeneic brainstem tumors in immunocompetent mice. The use of fully immunocompetent models was critical because we wished to investigate the interaction of immune-mediated therapies with tumor cells as well as with endogenous effectors of both the innate and adaptive immune system. For these studies, we used tumor lines which we have previously validated as optimally sensitive to the therapies that we tested – namely B16tk, GL261, B16-ova, GL261-QUAD, and B16-EGFRvIII. Although these are not pediatric brain tumor cells, they represent experimental scenarios where we are most likely to observe anti-tumor efficacy and therapy-related toxicity in an immunocompetent system.

Our first model was an optimal scenario for therapy in which 100% of the tumor cells were engineered to express the therapeutic HSVtk gene. Ours and others’ previous studies have shown that HSVtk/GCV tumor killing is highly inflammatory, generating both innate and adaptive immune responses [29, 30, 44]. Therefore, this first model was chosen to test the toxicity of massive tumor cell death associated with innate immune activation and subsequent T cell recruitment. Our data clearly show that drug-induced tumor killing in the brainstem was significantly more beneficial than toxic (Fig. 1B-D), despite the dramatic immune infiltration that was observed (Fig. 2). We also confirmed that current standard of care radiation therapy or dexamethasone could be effectively combined with this cytotoxic immunotherapy (Fig. 1B, D**)**. Dexamethasone co-treatment was also performed with oncolytic virus and T cell therapies, but had no significant effect on overall survival or therapeutic toxicity.

Our results here support the development of direct in vivo delivery of suicide genes such as HSVtk for the treatment of pediatric tumors such as DIPG. One promising drug delivery technique is convection enhanced delivery (CED), which was used in two recently completed clinical trials treating DIPG [45, 46]. While neither trial used immune-activating therapies, toxicity profiles were generally tolerable, with most neurologic symptoms resolving within four weeks after therapy. In light of these promising results, we are developing rodent models of convection enhanced delivery to test viral vectors expressing such genes.

The suicide gene therapy model demonstrated that inflammation associated with synchronized killing of tumors in the brainstem is not itself necessarily fatal. Therefore, we next tested oncolytic virotherapy, which we hypothesized would be significantly more inflammatory than HSVtk/GCV-mediated cell killing. In this respect, we have previously shown that oncolytic virotherapy is highly immunostimulatory through association with both tumor cell death and the presence of multiple viral immunogens and TLR activators [47]. Using two different oncolytic viruses, we again tested the best case scenario in which 100% of tumor cells were infected with either reovirus or with vaccinia virus (VV-GMCSF). Unlike treatment with HSVtk, we observed some acute, fatal toxicity with VV-GMCSF, with histologic analysis confirming the presence of a meningeal immune infiltrate (Fig. 3B,C). However, those mice which survived the toxicity were tumor free after 150 days, implying complete tumor clearance by either direct viral oncolysis, immune-mediated clearance, or both. Interestingly, mice injected with tumor cells pre-infected with reovirus did not develop similar catastrophic toxicity yet were also tumor free after 150 days. This efficacious response to reovirus in the absence of toxicity led us to perform subsequent studies of direct intratumoral injection into established tumors. In these studies, reovirus was neither more toxic nor therapeutic than control treatment (Fig. 3D). It remains a formal possibility that the reovirus did not infect each established GL261 tumor. However, we believe that this is unlikely based on several lines of evidence. In the first instance, our in vitro data (Fig. 3A) demonstrate that GL261 cells exposed to reovirus are readily infected and support reovirus replication. Second, injection of 2 μl of trypan blue into the brainstems of mice showed a diffuse and regionally comprehensive distribution, suggesting that even if the injection itself missed the tumor the tumor cells would be exposed to injected virus. Finally, because of the potential for variability in the injection procedure, we used identical stereotactic coordinates and operators for injection of both tumor cells and subsequent virus in three independent experiments, all of which showed the similar result that reovirus alone did not significantly increase survival.

Previous studies using oncolytic viruses to treat glioma [37, 38] led us to hypothesize that inhibitory receptors may be blunting the anti-tumor immune response, so we also added anti-CTLA-4 and anti-PD1 combination immune checkpoint blockade (ICB). Surprisingly, the addition of ICB did not cause observable toxicity, but increased survival beyond untreated controls (Fig. 3E). We have previously shown that systemic delivery of reovirus, in combination with GMCSF, led to therapy for both B16 and GL261 tumors in the temporal lobe of the brain and that addition of anti-PD1 ICB enhanced therapy [38]. Based on those and our current data, we are currently investigating the mechanisms by which reovirus replication, oncolysis, and immune activation are limited in established brainstem tumors following intratumoral injection and are testing systemic delivery of reovirus to the brainstem. In summary, our data show that the use of different viruses can be tolerated to different extents and support the cautious investigation of oncolytic virotherapy for brainstem tumors.

Both the suicide gene and oncolytic virus models of immunotherapy depend upon recruitment of potent innate immune responses prior to the development of adaptive T cell responses. Therefore, we went on to test the balance between toxicity and efficacy of therapies mediated directly by T cells. Importantly, our adoptive T cell transfer models required only systemic administration of T cells with no need for direct delivery of the therapeutic to the brainstem tumor itself. In our two different models of adoptive T cell therapy using transgenic and CAR T cells, we observed significant survival extension without overt symptoms of neurologic or systemic toxicity (Fig. 4-6**)**. We showed that therapeutic T cells trafficked to the tumor site and that therapy was dependent solely upon activation of the adoptively transferred T cells. Of particular importance, we did not observe overt signs of Cytokine Release Syndrome (CRS) or the related neurotoxicity frequently observed in clinical trials of CAR T cell therapy( [48, 49])This is in contrast to a study of CAR T cells against brainstem xenografts that demonstrated lethal toxicity in several mice, with frequency increasing in thalamic tumors [50]. This suggests that while the potential for CAR toxicity in the CNS exists, it is likely location-dependent and may be reduced by the presence of an endogenous immune system. Alternatively, CAR T cell adverse events have been shown to be related to tumor burden [51, 52], suggesting that treatment of smaller brainstem tumors may reduce CRS or neurotoxicity.

While these syngeneic models effectively model the host response to potentially toxic viro- or immunotherapies, they have several limitations that will inform future studies. Unlike the transplantable tumors that we used in our current studies, DIPG and other high grade gliomas are highly infiltrative, with tumor cells found in distant, otherwise normal tissue [53]. While we hypothesize that viroimmunotherapies are potentially capable of controlling this type of infiltrative disease due to diffusion of virus and migration of T cells [54, 55], this may also result in diffuse therapeutic toxicity. Future studies in genetically engineered mouse models will be important to assess both efficacy and toxicity in more infiltrative tumors.

Clinically it is important to investigate whether the therapeutic immune infiltrates that we observed in our studies here, with treatment starting against relatively small tumors, can also be tolerated in the context of symptomatic tumors. In this respect, we have found that mice in which GCV treatment was initiated just 2 days before tumors became lethal, still tolerated the therapy well and survived to similar time points as mice receiving much earlier therapy (8 days prior to tumor lethality).

Overall, these studies provide support for continued clinical investigation into viro- and immunotherapies for DIPG, particularly when combined with recent preliminary results from clinical trials. Treatment of DIPG patients in Phase I trials using DNX-2401 oncolytic adenovirus [56, 57], a DIPG lysate-loaded autologous dendritic cell vaccine [58], and an anti-PD1 antibody [59] all similarly indicate that the brainstem can tolerate immunotherapies, though clinical studies to date have not confirmed immune infiltrates or replicating virus, nor have they established anti-tumor efficacy. These clinical results stand in contrast to the pembrolizumab study for pediatric high grade glioma and DIPG, which encountered severe adverse events requiring temporary suspension (NCT02359565). Additionally, patients receiving CD19-reactive CAR T cells can exhibit neurotoxicity, though the mechanism of this event remains unclear [49]. This suggests that while ours’ and others’ studies suggest a generally favorable toxicity profile, there are still associated risks that may require detailed molecular analysis of tumors to predict those that will respond favorably versus those that will experience adverse events.

## Conclusions

In summary, our goal was to investigate whether immunotherapy for brainstem tumors was likely to be too dangerous to pursue. We screened four distinct models, each shown to be highly efficacious in models of peripheral tumor immunotherapy and associated with innate and adaptive components of the immune system. Of the four models tested, we observed significant toxicity only in VV-GMCSF treatment of murine gliomas in the brainstem. In all four models we were able to achieve anti-tumor efficacy without unmanageable toxicity. Our studies here were not designed to compare the relative efficacies of these different immunotherapies or to endorse any specific modality; such studies will require the development of efficient in vivo delivery systems, careful testing of different vectors, transgenes, and T cell targets, and development of genetically engineered, spontaneous glioma models to complement the studies described here. Despite the positive nature of our results showing that immunotherapies can be effective, in the light of other preclinical and clinical studies discussed above, we believe that it remains absolutely imperative to regard the brainstem as a site of concern for immunotherapeutic intervention. Nonetheless, further careful development of immunotherapies for pediatric brainstem tumors is warranted to harness the potential potency of innate and adaptive anti-tumor immune responses, while limiting their possible toxicity within this anatomically sensitive location.

## Data Availability

Not applicable.

## Abbreviations

B16tk: B16 murine melanoma expressing herpes simplex virus thymidine kinase
CAR: Chimeric antigen receptor
DIPG: Diffuse intrinsic pontine glioma
EGFRvIII: Epidermal growth factor receptor variant III
GCV: Ganciclovir
Ova: Ovalbumin
PBS: Phosphate buffered saline
TBI: Total body irradiation
UTD: Untransduced
VSV: Vesicular stomatitis virus
VV-GMCSF: Vaccinia virus encoding granulocyte monocyte colony stimulating factor
